# Anorexia nervosa – medical complications

**DOI:** 10.1186/s40337-015-0040-8

**Published:** 2015-03-31

**Authors:** Philip S Mehler, Carrie Brown

**Affiliations:** Department of Medicine, University of Colorado Health Sciences Center, ACUTE at Denver Health, and Eating Recovery Center, Denver, CO – 777 Bannock Street, MC4000, Denver, CO 80204, 7351 E Lowry Blvd, Suite 200, Denver, CO 80230 USA; Department of Medicine, University of Colorado Health Sciences Center and ACUTE at Denver Health, Denver, CO – 777 Bannock Street, MC4000, Denver, CO 80204 USA

**Keywords:** Anorexia nervosa, Medical, Complications, Gastrointestinal, Cardiac, Osteoporosis

## Abstract

In contrast to other mental health disorders, eating disorders have a high prevalence of concomitant medical complications. Specifically, patients suffering from anorexia nervosa (AN) have a litany of medical complications which are commonly present as part of their eating disorders. Almost every body system can be adversely, affected by this state of progressive malnutrition. Moreover, some of the complications can have permanent adverse effects even after there is a successful program of nutritional rehabilitation and weight restoration. Within this article we will review all body systems affected by AN. There is also salient information about both, how to diagnose these medical complications and which are the likely ones to result in permanent sequelae if not diagnosed and addressed early in the course of AN. In a subsequent article, the definitive medical treatment for these complications will be presented in a clinically practical manner.

## Background

Anorexia nervosa and bulimia are both inherently associated with many different medical complications. This review article is part one of a planned three part series of articles in this regard. We will focus solely on the medical complications associated with restricting anorexia nervosa. Part two of this series will be devoted to the medical complications associated with bulimia nervosa and, the third paper will discuss the treatments currently available for the medical complications of both anorexia nervosa and bulimia. Some of this information is based on experienced clinical opinion.

Anorexia nervosa is associated with numerous general medical complications [1]. The complications affect almost all major organ systems and often also include physiologic disturbances such as hypotension, bradycardia and hypothermia. Medical complications account for more than half of all deaths in patents with anorexia nervosa [2]. Standardized mortality ratios show that the rate of death in anorexia nervosa is 10 to 12 times greater than the rate in the general population [3,4]. Indeed, anorexia nervosa has the highest mortality rate of any psychiatric disorder, likely due to these medical complications.

In general, medical complications of anorexia nervosa are a direct result of weight loss and malnutrition. Starvation induces protein and fat catabolism that leads to loss of cellular volume and function, resulting in adverse effects on, and atrophy of, the heart, brain, liver, intestines, kidneys, and muscles [Table 1].

**Table 1:** **Medical complications of anorexia nervosa**

**Cardiovascular**	**Endocrine and Metabolic**
Bradycardia and hypotension	Amenorrhea
Mitral valve prolapse	Infertility
Sudden death - arrhythmia	Osteoporosis
Refeeding syndrome	Thyroid Abnormalities
Echo changes	Hypercortisolemia
**Dermatologic**	Hypoglycemia
Dry skin	Neurogenic diabetes insipidus
Alopecia	Arrested growth
Lanugo hair	**Hematologic**
Starvation-associated pruritis	Pancytopenia due to starvation
**Gastrointestinal**	Decreased sedimentation rate
Constipation	**Neurologic**
Refeeding pancreatitis	Cerebral atrophy
Acute gastric dilatation delayed gastric emptying	**Opthalmic**
Hepatitis	Lagopthalmos
Dysphagia	**Pulmonary**
	Aspiration pneumonia
	Respiratory failure
	Spontaneous pneumothorax
	Emphysema

The reported incidence of these medical complications varies, depending upon the individual patient and also on the severity of the episode of anorexia nervosa. The primary risk factors for developing medical complications in anorexia nervosa are the degree of weight loss and the chronicity of the illness [5]. There are no known sociodemographic risk factors for developing complications.

### Dermatological

As weight loss worsens due to the nutritional deprivation, it is common for the patient with anorexia nervosa to have dry skin which can fissure and bleed especially in the fingers and toes [6]. Also it is common for these patients to have cold intolerance and a bluish discoloration to the distal tips of their fingers as well as their nose and ears. This is referred to as acrocyanosis, and may be due to the shunting of blood flow centrally in response to the hypothermia seen with anorexia nervosa. Lanugo hair growth, which is fine downy hair on the sides of the face and along the spine, is regularly noted with anorexia nervosa and may represent an attempt by the body to conserve heat. Decubitus ulcers over boney prominences may develop due to loss of supporting subcutaneous tissue and needs to be looked for at the time of physical examination because delayed wound healing is also part of the cutaneous manifestations of starvation. Easy bruisabilty is likewise related to the relative absence of subcutaneous tissue due to weight loss.

### Gastrointestinal

With pure food restriction, once weight loss below approximately 15–20 percent of ideal body weight occurs, there is often the development of gastroparesis [7]. Gastroparesis refers to delayed emptying of the stomach. Bloating, upper quadrant pain and early satiety are the main symptoms, and may be severe. Acute gastric dilation, which should be screened for with an abdominal X-ray if the patient complains of severe left upper quadrant pain or has significant vomiting, is an extreme and rare result of delayed gastric emptying. If this diagnosis is missed, the massive dilatation of the stomach can result in gastric necrosis, perforation and death [8,9]. Bloating can be worsened by a high-fiber diet that these patients may resort to in an attempt to treat their slowed gastrointestinal transit. In rare cases it may be necessary to obtain a nuclear medicine gastric emptying study to investigate prolonged symptomatology.

Similarly, constipation commonly accompanies the weight loss of anorexia nervosa. Patients may complain of bowel movements that are infrequent or small. It is helpful from the start to reassure these patients that bowel patterns in healthy patients may normally vary anywhere from two times per day to just a few times per week, and that persons with anorexia nervosa issues are expected to indeed have even fewer bowel movements. Constipation in these patients is due either to drastically reduced caloric intake, which results in reflex hypofunctioning of the colon, or to slow colonic transit.

An upright abdominal X-ray may be useful to exclude abnormal bowel distention when symptoms of constipation persist after an adequate trial of interventions aimed at alleviating constipation. The absence of excessive stool on these radiographic studies provides the clinicians caring for these patients and the patients with proof that bowel function is normal and no longer deserves ongoing concern. This is especially helpful because the interplay of functional gastrointestinal disorders is significantly prevalent in patients with anorexia nervosa [10], in the form of the irritable bowel syndrome.

Liver transaminases (AST & ALT) are often abnormal in anorexia nervosa, occurring in almost half of all patients with anorexia nervosa [11]. Weight loss and fasting can produce mild elevation (2-3x normal) of transaminases (AST/ALT). Mild transaminase elevation can also occur early in the course of refeeding if dextrose calories are excessive, and is referred to as steatosis. These elevations usually resolve and normalize if the daily caloric intake and the amount of dextrose calories are temporarily decreased. A higher level of calorie intake can then be reintroduced at a later date once the liver tests have normalized. The transaminase may also be markedly elevated (4-30x normal) with severe anorexia nervosa, even before refeeding has started, and may be a sign of serous multiorgan failure [12]. Nutritional support will usually result in improvement. If the liver function tests are elevated during the early phases of refeeding, a liver ultrasound can help distinguish starvation-induced enzyme elevations from refeeding-induced elevations. During starvation, the ultrasound typically reveals that the liver is small in size whereas the ultrasound in refeeding hepatitis the ultrasound may show an enlarged fatty liver [13]. The starvation-induced elevations are more likely to occur in patients with a body mass index (BMI) less than 12/kg/m^2^ [14]. The exact cause of this phenomenon is not known. Putative causes include autophagy or organ hypoperfusion due to the myocardial dysfunction seen in anorexia nervosa.

Another gastrointestinal complication to be aware of in patients with anorexia nervosa is the superior mesenteric artery syndrome (SMA). It results from compression of the duodenum between the aorta and spine posteriorly and the SMA anteriorly as a result of loss of the adipose tissue fat pad that normally surrounds the SMA, as a direct result of weight loss. This narrows the angle between the two blood vessels and entraps the duodenum. The SMA syndrome manifests with upper quadrant abdominal pain soon after eating along with early satiety, nausea and vomiting. Abdominal CT scan or an upper GI series are diagnostic and reveal an abrupt cut-off of the third portion of the duodenum as it passes between the SMA and aorta [15].

Aspiration of oral calories into the airways, both liquids and solids, may also occur in more severe cases of anorexia nervosa due to dysphagia that is caused by pharyngeal muscle weakness resulting from protein-calorie malnutrition [16]. Difficulty swallowing and uncoordinated transfer of the food bolus from the mouth to the stomach may lead to aspiration and even result in aspiration pneumonia. A bedside swallow evaluation by a speech therapist and/or a video fluoroscopic swallow study can confirm the diagnosis. If dysphagia and aspiration are confirmed, modifying the consistency of foods or inserting a temporary feeding tube may be required until sufficient weight gain restores normal swallow function.

Acute pancreatitis in patients with anorexia nervosa is rare, but has been described during refeeding [17]. The presumptive etiology is that malnutrition activates proteases such as trypsin which injures pancreatic cells. Its presentation, during the early phases of refeeding, is typical for pancreatitis and is characterized by epigastric pain which radiates posteriorly, accompanied by nausea and vomiting and associated with elevations of the pancreatic enzymes amylase and lipase.

### Endocrine

Patients with anorexia nervosa have a number of abnormalities in endocrine function. Secretion rates of cortisol are generally elevated [18], and metabolic clearance rates are decreased, with the result that the half-life of cortisol may be prolonged in malnourished individuals. The clinical significance of this elevated cortisol level is unknown, but it may be involved with loss of bone density in anorexia nervosa.

Alterations in growth hormone are also present in anorexia nervosa. Levels are more often elevated, but levels of insulin like growth factor (IGF-1) are decreased, indicative of growth hormone resistance [19]. The clinical significance of this finding is not clear. Antidiuretic hormone levels may also be low in anorexia nervosa which may rarely result in central diabetes insipidus manifested by hpernatremia.

The thyroid abnormalities in individuals with anorexia nervosa resemble those of the euthyroid sick syndrome, in which total thyroxine (T_4_) and triiodothyronine (T_3_) levels are low. The key, however, is that thyroid stimulating hormone (TSH) usually remains in the normal range [20]. Levels of T_3_ usually decrease in proportion to the degree of weight loss. Total T_4_ levels are low because T_4_ is preferentially converted to a biologically inactive reverse T_3_. It is important to avoid unnecessary and potentially dangerous thyroid hormone replacement therapy for low-weight anorexic patients with the aforementioned thyroid function test findings because these alterations in thyroid function tests, normalize with nutritional rehabilitation. The risks of unnecessary thyroid hormone are especially prominent both because of its deleterious effect on bone mineral density, in a population of patients who are already at risk for severe osteoporosis, and because of its effect to increase metabolic rate and frustrate weight gain.

Dietary restriction accompanied by weight loss and excessive exercise lead to depletion of hepatic glycogen stores and disruption of hepatic gluconeogenesis, resulting in abnormalities of glucose metabolism and hypoglycemia. In milder cases of anorexia, hypoglycemia is not generally present. In contrast, individuals with advanced anorexia nervosa develop hypoglycemia [21]. Severe hypoglycemia has been associated with sudden death because it indicates liver failure and a depletion of substrate to maintain safe blood glucose levels [22]. In the presence of hypoglycemia, insulin levels are appropriately decreased in anorexia nervosa. Recent studies indicate that individuals who are older have a higher risk of hypoglycaemia [23]. Rare reports of reactive hypoglycemia during early refeeding have also been reported in anorexia nervosa [24].

Anorexia nervosa is occasionally complicated by comorbid Type 1 Diabetes Mellitus. While the exact casual association between type 1 diabetes mellitus and anorexia nervosa has not been fully elucidated, these two disorders do sometimes coexist in the same patient. This in turn creates treatment challenges, especially during the early phases of refeeding and is associated with an increased mortality risk [25].

It is irrefutably clear that excessive hyperglycemia and poor glucose control, in all diabetic patients, are associated with premature microvascular complications such as diabetic retinopathy and nephropathy [26]. One can however logically posit that this concern is only relevant over the course of the lifetime of a patient with type 1 diabetes mellitus. It is not likely to be of clinical significance if present for a period of just a few weeks during a structured refeeding program for the diabetic patient with severe anorexia nervosa, as long as his or her level of hyperglycemia is not excessive (i.e., glucose level less than 250 mg/dL). Thus, an allowance for “permissive hyperglycemia,” is certainly more conducive to building the requisite therapeutic trust which is so critical in the refeeding program of a patient with anorexia nervosa. This approach should be followed during the early stages of refeeding as opposed to a weight-restored state where tight glucose control is again sought [27].

Sex hormones are affected in both male and female patients with anorexia nervosa. These patients have low levels of hypothalamic gonadotropin releasing hormone (GnRH) and low levels of pituitary luteinizing (LH) and follicle stimulating hormone (FSH), estrogen and testosterone. These abnormalities affect potency, fertility and bone density. The neuroendocrine regulation of normal female reproductive functions depends on a rhythm of nerve impulses generated within the medial basal hypothalamus, which governs the pulsatile release of GnRH from nerve terminals. Pulsatile GnRH release is the central controller of pituitary LH and FSH secretion, which determine the time onset of normal menstrual function [28]. Patients with anorexia nervosa reproducibly have a characteristic “hypothalamic amenorrhea syndrome” with a variable reduction in pulsatile hypothalamic GnRH gonadostat signalling to the pituitary gland, resulting in a failure of ovulation. The degree of impairment varies among patients with anorexia nervosa, but in general, the frequency and amplitude of the LH-FSH pulses are diminished, with a reversion to a prepubertal pattern and the development of the commonly found amenorrheic state. Thus, this functional amenorrhea seen in anorexia nervosa reflects a temporary, reversible disturbance of hypothalamic-pituitary function. Most amenorrhea seen with anorexia nervosa is of the secondary type, meaning the patient previously had normal menstrual periods.

Of patients with anorexia nervosa, 20–25 percent may experience amenorrhea before the onset of significant weight loss, and 50–75 percent will experience amenorrhea during the course of dieting and its weight loss [29]. In some patients with anorexia nervosa, amenorrhea occurs only after more marked weight loss [30]. Overall, the development of amenorrhea is most strongly correlated to loss of body weight. As a result of the aforementioned changes in reproductive hormones, patients with anorexia nervosa have difficulty conceiving, but, importantly, patients with anorexia nervosa may ovulate and become pregnant despite their amenorrhea. Unplanned pregnancy is a risk in anorexia nervosa [31]. Overall, the incidence of infertility is increased in anorexia nervosa due to the commonly found amenorrhea and decreased libido. If pregnancy does occur, there is also a higher rate of pregnancy complications as well as neonatal complications [32]. Increased numbers of miscarriages have also been reported in anorexia nervosa [33].

### Hematologic

The bone marrow is adversely affected by anorexia nervosa. All three cell lines, namely red blood cells, white blood cells and platelets, may be affected by anorexia nervosa. Specifically, anemia and leukopenia occur in approximately one-third of the patients and thrombocytopenia occurs in ten percent [34]. The basic pathology of the affected marrow demonstrates a hypoplastic marrow with gelatinous deposition and serous fat atrophy [35]. As disease severity worsens and BMI falls, the frequency of these abnormalities is greater with upwards of seventy-five percent of patients demonstrating cytopenias [36]. However, there is no characteristic change in red cell size with most patients having normal indices. Similarly, all white cell types are proportionately reduced to cause neutropenia and lymphopenia, but no consistent pattern emerges for anorexia nervosa. The serum international normalized ratio (INR) level may be mildly elevated, due to liver damage and impaired synthesis of coagulation factors; patients may thus present with petechiae and purpura [37].

Interestingly, patients with anorexia nervosa do not seem to be predisposed to more frequent infectious diseases, notwithstanding their malnourished states. However, because the usual signs of infection (fever and elevated white blood cell count) may not be present in anorexia nervosa, increased vigilance and a lower threshold to evaluate for an infection should be followed [38].

### Neurologic

Recent studies have demonstrated that anorexia nervosa is associated with variable, but usually significant, brain atrophy [39]. Severe cases of anorexia nervosa may appear, on magnetic resonance imaging (MRI), to be indistinguishable from the brain of a person with Alzheimer’s disease; ventricles are enlarged and cortical substance is decreased [40]. While anorexic patients often have a surprising degree of accomplishment in school, as weight erodes they become increasingly unable to attend to, and concentrate on, written materials or sustain reasoning. Of concern is the recent demonstration that weight improvement is not immediately associated with complete restoration of normality in the MRI brain scan, especially of the gray matter. This may be correlated with the duration of illness as recent studies from adolescents with a history of anorexia nervosa, when weight restored, have not revealed global or regional gray or white matter abnormalities [41]. Work is under way with positron emission tomography (PET) to localize the specific brain regions most affected by starvation so as to determine their response to treatment. Of note, there are no consistent peripheral nerve findings associated with anorexia nervosa, although with more marked weight loss comes overall weakness and deconditioning.

### Bone metabolism

Patients with anorexia nervosa very commonly have impaired bone structure and reduced bone strength. Various modalities exist for assessment of bone density. Dual X-ray absorptiometry (DEXA) is the most commonly-used modality and measures the bone mineral content for a given cross sectional area of bone. Using DEXA scan, a T-score, which reflects a young adult population, and Z-score, which reflects an age-matched population, are determined. The World Health Organization defines osteoporosis in postmenopausal women as a BMD value at the spine, hip, or forearm of 2.5 or more standard deviations (SD) below the young adult mean (T-score ≤ −2.5). Osteopenia is defined as a T-score between −1 and −2.5 [42]. Definitions for bone density loss among young, pre-menopausal women and men have not been officially defined, however, measurement of bone density remains of great utility in patients with anorexia nervosa. MRI has been used to determine marrow fat content and composition among patients with anorexia. Higher marrow fat inversely correlates with bone mineral density [43].

In fact, 85% of women with a diagnosis of anorexia nervosa have either osteoporosis or osteopenia [44]. A study of 310 women showed lifetime fracture prevalence being 60% higher in those with anorexia nervosa as compared to controls [45]. Individuals who develop anorexia during adolescence are especially of great concern as bone accrual continues normally through the mid-20s and thus these individuals may never reach normal peak bone mass. Women who develop anorexia nervosa as adolescents, end up having lower bone mineral density than women who develop anorexia nervosa during adulthood with similar duration of amenorrhea [46].

This low bone mass is due to reduced bone formation and increased bone resorption. Multiple hormonal adaptations, designed to decrease energy expenditure during periods of low energy intake, may be to blame for this phenomenon. The aforementioned elevated growth hormone (GH) levels may be important for mobilizing fat stores in the setting of nutritional deprivation. IGF-1 mediates the actions of GH on bone metabolism. Low IGF-1 levels may decrease energy expenditure among several physiologic processes in the body, including the maintenance of bone mass. Also, similar to the effects of estrogen deficiency in postmenopausal women, this deficiency found in anorexia nervosa, due to the ubiquitous hypogonadotropic hypogonadism of anorexia nervosa, results in an increase in bone resorption and decreased bone mass [44]. According to one study, duration of amenorrhea in anorexia nervosa was the only factor associated with decreased lumbar spine bone mineral density and IGF-1 levels were the only significant independent predictor of decreased bone mineral density (BMD) of the proximal femur [47].

Males with anorexia nervosa also have osteopenia and osteoporosis as noted above. Charts from 70 consecutive males treated for anorexia nervosa revealed 36% had osteoporosis and 26% had osteopenia at the lumbar spine. Lower BMI and longer illness duration predicted lumbar Z-scores [44]. Low testosterone levels may also correlate directly with degree of bone mineral density loss [48]. In fact, male patients with anorexia nervosa seem to have worse degrees of low bone density compared with female anorexia nervosa patients [49].

### Cardiac

Bradycardia (pulse <60) and hypotension are among the most common physical findings in patients with anorexia nervosa, with bradycardia seen in up to 95% of patients. Anorexia nervosa should be considered in the differential for unexplained bradycardia in the outpatient setting [50]. In addition, resting tachycardia is highly unusual and may be indicative of a superimposed infection or other complication [51]. Heightened vagal tone has been suggested as the cause of bradycardia in the setting of anorexia nervosa [52]. Low blood pressure and heart rate universally increase to normal levels after refeeding and restoration of normal weight [53].

Structural abnormalities, including pericardial effusion and decreased left ventricular size are also commonplace in the setting of anorexia nervosa. Silent pericardial effusion is present in 22% to 71% of patients with anorexia nervosa by echocardiography [54-56]. Factors which may correlate with pericardial effusion in this patient population include low BMI, rapid weight loss, low T_3_ levels, and IGF-1 levels [57]. Most patients show resolution of the effusion after weight restoration without further intervention necessary; however, there are case reports of cardiac tamponade and the rare need for urgent pericardiocentesis for prevention thereof [58,59].

Multiple studies of patients with anorexia nervosa have revealed findings of decreased left ventricular mass, left ventricular index, cardiac output, and left ventricular diastolic and systolic dimensions [56]. Longstanding hypovolemia has been postulated as a potential cause for these findings [60]. Mitral valve motion abnormalities, including mitral valve prolapse, may also be seen in a distinct minority. This can cause chest pain and palpitations in these patients. However, the ejection fraction appears to remain preserved in most cases [54]. Weight restoration has also been shown to correlate with normalization of cardiac dimensions [61].

Beyond bradycardia, more subtle arrhythmias have the potential to create significant complications for patients with anorexia nervosa. The QT interval, as measured on electrocardiogram (ECG), is commonly used in cardiology as a marker for arrhythmogenicity. QT dispersion, or the difference between maximum QT interval and minimum QT interval on ECG, is another concerning marker when increased. Prolonged QT and increased QT dispersion may also indicate that the patient is at risk for sudden cardiac death [62]. Increased QT interval and QT dispersion among patients with anorexia have been reported in the literature [63,64]. However, some studies have linked QT prolongation to hypokalemia and increased vagal activity and not intrinsically related to anorexia nervosa [65]. Prolonged QT has thus not been suggested as an inherent marker for disease severity in anorexia nervosa as many confounding factors exist, including prolongation due to commonly-prescribed medications such as antipsychotic medications. Weight restoration has been shown to resolve findings of prolonged QT and QT dispersion [61,53]. It should be noted that there are case reports of an extremely rare cause of reversible acute heart failure, known as takotsubo cardiomyopathy, among patients with anorexia. This condition has previously been linked to prolonged QT interval [66,62] but is more commonly related to elevated catecholamine levels due to severe psychological or physical stress.

### Pulmonary

The lungs are not immune to the adverse effects of anorexia nervosa and malnutrition as was once thought. Multiple case reports now show findings of emphysema on imaging among patients with anorexia nervosa, even without a smoking history [67,68]. Diffusion capacity of the lung for carbon monoxide (DLCO) and lung diffusion capacity for oxygen have been shown to progressively worsen with anorexia nervosa disease duration [69]. Findings of emphysema and decreased pulmonary function are by no means universal among patients with anorexia nervosa [70].

Two potentially life-threatening, albeit rare complications, of anorexia nervosa include pneumothorax and pneumomediastinum. These are infrequently known to occur spontaneously among patients with anorexia [71,72] and may pose significant difficulties with management [73]. Spontaneous tension pneumoperitoneum and tension pneumothorax have also been reported as resulting from acute gastric rupture in eating disorder patients who both restrict and purge via self-induced vomiting [74].

### Males with anorexia nervosa

The very starved male and female patients are similar medically with the exception that males start with a lower reserve percentage of body fat and a higher lean muscle mass, allowing him less weight loss before the onset of ketosis and protein breakdown. In contrast to the occurrence of amenorrhea in females, males have no comparable “signal” that alerts family to the medical consequences of weight loss. In addition, boys and men who suspect they may have an eating disorder often perceive, quite accurately, stigma from society, from eating-disordered females, and from peers. Thus, they may be hesitant to discuss this possibility with clinicians. Therefore, they often present after more severe weight loss and with more extensive clinical and laboratory findings [75].

Their history will include changes in sexual functioning, including a decrease in sexual drive. Physical exam will note the general degree of emaciation and decline in lean muscle mass, as well as the aforementioned general medical findings including vital sign changes. Laboratory studies in the male should include serum testosterone level. Testosterone declines in proportion to weight loss. LH and FSH will be correspondingly diminished in anorexia nervosa because the changes in gonadotropins are due to central hypothalamic hypogonadism secondary to starvation, rather than increasing as would be expected with a failing gonad. Testicular examination will often reveal testes that are small.

## Conclusions

In summary, anorexia nervosa has a litany of medical complications which are associated with it. The general rule is that they become more apparent as the patient’s weight falls further from normal. Most body systems can be adversely affected. However, the encouraging message is that the vast majority of these, often serious medical complications, are reversible with weight gain and nutritional rehabilitation as will be described in the third segment of this series.

## Abbreviations

ALT: Alanine transaminase
AN: Anorexia nervosa
AST: Aspartate aminotransferase
BMD: Bone mineral density
BMI: Body mass index
CT: Computed tomography
DEXA: Dual x-ray absorptiometry
DLCO: Diffusion capacity of the lung for carbon monoxide
ECQ: Electrocardiogram
FSH: Follicle stimulating hormone
GI: Gastrointestinal
GH: Growth hormone
GnRH: Gonadotropin releasing hormone
IGF-1: Insulin like growth factor
INR: International normalized ratio
LH: Luteinizing hormone
MRI: Magnetic resonance imaging
PET: Position emission tomography
SD: Standard deviation
SMA: Superior mesenteric artery
T_3_: Thyroxine
T_4_: Triiodothyronine
TSH: Thyroid stimulating hormone
